# A productive clash of perspectives? The interplay between articles’ and authors’ perspectives and their impact on Wikipedia edits in a controversial domain

**DOI:** 10.1371/journal.pone.0178985

**Published:** 2017-06-02

**Authors:** Jens Jirschitzka, Joachim Kimmerle, Iassen Halatchliyski, Julia Hancke, Detmar Meurers, Ulrike Cress

**Affiliations:** 1 Leibniz-Institut für Wissensmedien, Tübingen, Germany; 2 Department of Psychology, Eberhard Karls University of Tübingen, Tübingen, Germany; 3 Department of Linguistics, Eberhard Karls University of Tübingen, Tübingen, Germany; Nanjing Normal University, CHINA

## Abstract

This study examined predictors of the development of Wikipedia articles that deal with controversial issues. We chose a corpus of articles in the German-language version of Wikipedia about alternative medicine as a representative controversial issue. We extracted edits made until March 2013 and categorized them using a supervised machine learning setup as either being *pro conventional medicine*, *pro alternative medicine*, or *neutral*. Based on these categories, we established relevant variables, such as the perspectives of articles and of authors at certain points in time, the (im)balance of an article’s perspective, the number of non-neutral edits per article, the number of authors per article, authors’ heterogeneity per article, and incongruity between authors’ and articles’ perspectives. The underlying objective was to predict the development of articles’ perspectives with regard to the controversial topic. The empirical part of the study is embedded in theoretical considerations about editorial biases and the effectiveness of norms and rules in Wikipedia, such as the neutral point of view policy. Our findings revealed a selection bias where authors edited mainly articles with perspectives similar to their own viewpoint. Regression analyses showed that an author’s perspective as well as the article’s previous perspectives predicted the perspective of the resulting edits, albeit both predictors interact with each other. Further analyses indicated that articles with more non-neutral edits were altogether more balanced. We also found a positive effect of the number of authors and of the authors’ heterogeneity on articles’ balance. However, while the effect of the number of authors was reserved to pro-conventional medicine articles, the authors’ heterogenity effect was restricted to pro-alternative medicine articles. Finally, we found a negative effect of incongruity between authors’ and articles’ perspectives that was pronounced for the pro-alternative medicine articles.

## Introduction

Online communities like Wikipedia provide platforms where knowledge is efficiently developed in a complex process of mass collaboration [1–2] and peer production [3]. Large numbers of people with diverse background knowledge and points of view may be involved in this endeavor [4]. They coordinate their joint writing process often only indirectly through the article texts that they produce by adding, changing, and deleting information [5–6].

A good example for the promising potential of collaborative work in Wikipedia is the German-language Wikipedia article on the Fukushima Daiichi Nuclear Power Plant, which Oeberst, Halatchliyski, Kimmerle, and Cress [4] analyzed from March 11, 2011, when the Tōhoku earthquake and the following tsunami damaged the power plant, to March 19, 2011. Within this period of observation, amounts of new information were incorporated into the article, whereas biased or unverified information was modified or deleted very fast. Finally, the article version from March 19, 2011, was assessed by independent nuclear power plant experts and found to be high in quality and correctness. Oeberst et al. [4] interpreted this result as a consequence of the Wikipedia rules of *neutral point of view* (NPOV) and *verifiability*.

These policies ensured that the articles developed in an unbiased and objective direction. The NPOV description in Wikipedia contains, for example, the following remark: “Editors, while naturally having their own points of view, should strive in good faith to provide complete information, and not to promote one particular point of view over another” (Retrieved April 8, 2016, from https://en.wikipedia.org/wiki/Wikipedia:Neutral_point_of_view). Such norms and rules are determined, among others, by the fact that the broad spectrum of topics in Wikipedia covers many controversial issues. Accordingly, much research about Wikipedia focuses on controversies, for example, with regard to the automatic detection of controversies and disputes (e.g., [7–15]).

Wikipedia articles aim to be a collaborative product of many authors with different backgrounds, interests, knowledge, and opinions. Norms and rules such as the NPOV in Wikipedia are intended to take different perspectives into consideration and to prevent editorial biases. At least two related biases are worthy of note with regard to editorial biases: the *evaluation bias* and the *selection bias*. Whereas the evaluation bias describes the devaluing of attitude-inconsistent information and the overvaluing of attitude-consistent information (e.g., [16–20]), the selection bias describes another type of *confirmation bias*—namely, the selective search for and exposure to attitude-consistent information and the avoidance of attitude-inconsistent information (e.g., [21–24]; related phenomena are described, for example, as “echo chambers” [25], virtual “filter bubbles” [26], and “cyber-balkanization” of the Web [27]). Different and often incompatible views of the authors, that is, their different *author*-*polarities* in a two-sided issue (e.g., their stand on a pro-con dimension), sometimes even cause so-called “edit wars” that manifest themselves in frequent mutual revisions (e.g., elimination of others’ contributions) or even in vandalism [28–29].

However, the NPOV rule expects authors to write in a non-polarized way and to be open to the different perspectives that come along with a particular domain (see [30]). The consequences of such rule-guided collaboration should be apparent in the artifacts’ attributes, for example, whether Wikipedia articles become more or less neutral through the participation of many authors and their edits [31–32]. Such research typically shows that Wikipedia articles tend to possess a higher quality and become more neutral over time (e.g., [33–34]). In this regard, the number of edits and the number of unique authors per article could be seen as important attributes of high quality [35].

But controversies are not only caused by different interests or convictions of the authors. They are also often inherent in the topics themselves [10] because these may be quite complex, multifaceted, and many-sided. Moreover, controversies in Wikipedia may not necessarily lead to damaging or destructive processes. Some kind of moderate controversy or disagreement may be even necessary in order to stimulate a collective of individuals to construct new knowledge [36–39]. For example, by analyzing the Wikipedia pages of two domains (psychology and education) with methods of social network analysis (SNA), Halatchliyski and Cress [40] as well as Halatchliyski, Moskaliuk, Kimmerle, and Cress [41] have shown that new knowledge (new edits, new neighboring articles, edits in neighbors) did not only occur in the most central articles of each domain, but also in the border-crossing articles that linked both domains, that is, at the location where authors from different camps meet.

Thus, controversies may be necessary and productive: being confronted with arguments that contradict one’s own perspective can motivate an individual to reflect on and modify one’s own position. In the co-evolution model, Cress and Kimmerle ([36–37]; see also [38–39]) elaborated on this assumption and applied it to the interaction between wiki users and the digital artifacts. In this respect, Moskaliuk, Kimmerle, and Cress ([42–43]; see also [44]) have shown that the incongruity between an individual’s perspective and that presented in a wiki has a significant impact on what people contribute to the wiki and on how they develop their own convictions during the editing process. These findings, however, did not result from real collaboration on the Web, but from experimental laboratory settings where participants worked on simulated wikis. The studies systematically varied the level of incongruity between a participant’s perspective and that presented by the article. The results showed that people were most willing to adopt a new perspective if there was a medium level of incongruity between their perspective and that presented in the article. Their edits also improved the article with a medium level of incongruity the most, because in this case they tended to contribute to the wiki in such a way that the article considered both the pro- and con-arguments and weighted them in a more equivalent fashion.

In sum, these studies showed that a medium level of incongruity, that is, a *moderate* clash of divergent views, was the most productive for the further development of perspectives among both the contributors and the articles. Accordingly, it could be indeed beneficial for an article’s development if authors decided to contribute to an article moderately incongruent with their own point of view. But especially with regard to the selection biases mentioned above, it remains an open research question whether authors in natural settings actually prefer to contribute to articles with a medium level of incongruity. A related further question is: how does an author’s previous perspective influence his or her subsequent contributions to an article? And how does an article’s dominant perspective influence an author’s contributions to that article? Another open question is whether the dominant perspective of an article at a given point in time has an impact on how the article will develop further in the future.

### This study

This study is based on the considerations above and on previous empirical results. We analyzed the ways in which the ongoing development of Wikipedia articles was determined by articles’ and authors’ previous perspectives on an issue, the authors’ heterogeneity with respect to these perspectives, and the level of incongruity between the perspective of an author and that expressed in the current version of the Wikipedia article.

We assumed (a) that single edits can be classified as either neutral or non-neutral, (b) that non-neutral edits can be assigned to one of the two poles of a two-sided controversy (i.e., pros and cons), (c) that an article can be more or less unbalanced in one of these two directions, and (d) that the overall perspective of an article, that is, an article’s *polarity*, can be deduced from the proportion of its opposing non-neutral edits. This average polarity of an article describes the position of an article on a bipolar continuum in a two-sided controversy, with a balance midpoint in the middle of this continuum. Based on this, an article’s *imbalance* can be defined as the absolute value of an article’s polarity deviation from the continuum’s midpoint. To identify friction among different perspectives as imbalance predictors, we looked (a) at the difference between an article’s previous perspective and the author’s previous perspective (article-author-*incongruity*) and (b) at the differences among the authors’ perspectives who had edited an article previously (authors’ *heterogeneity*).

Our main focus was thereby not on neutral single edits but on the entirety of an article’s non-neutral edits and the proportion of opposing non-neutral edits. This perspective corresponds to the statements that the NPOV criterion can be fulfilled by striving for balanced articles in such a way that “biased information can usually be balanced with material cited to other sources to produce a more neutral perspective” and that “although specific article structures are not, as a rule, prohibited, care must be taken to ensure that the overall presentation is broadly neutral” (Retrieved April 8, 2016, from https://en.wikipedia.org/wiki/Wikipedia:Neutral_point_of_view).

We measured articles’ and authors’ perspectives by analyzing the semantics of substantial changes to the article text (see below for details). As a controversial domain, we chose a corpus of articles about alternative medicine in the German-language version of Wikipedia. Alternative medicine, by definition, contrasts with conventional medicine. We employed computational linguistic algorithms in order to classify changes of the articles toward a conventional or an alternative medical perspective as they were expressed in individual edits. Based on these classifications, we built *profiles of articles as well as of authors* (e.g., see [13], p. 175) that represented their current perspective (articles’ and authors’ *polarities*) on alternative medicine. This methodological procedure allowed us to determine and explain the distribution of the articles’ perspectives and their temporal development.

### Hypotheses

Against the empirical and theoretical background outlined so far, we state the following hypotheses on the *level of edits*, where articles’ and authors’ perspectives encounter each other:

H1: The incongruity between the authors’ current perspectives and the articles’ current perspectives will determine the articles’ development in such a way that there will be more non-neutral edits with a medium level of incongruity.

However, since we do *not* assume that robust phenomena like the confirmation bias will disappear completely within Wikipedia knowledge-construction processes, we state—in addition—the following hypotheses:

- H2: The current perspective of an author will predict the perspective expressed in future edits by this author, whereby both should point in the same direction.
- H3: The current perspective of an article will predict the perspective expressed in future edits in this article, whereby both should point in the same direction.

On the *article level*, we focus on the articles’ imbalance and postulate the following hypotheses:

- H4: Articles with more non-neutral edits will be less unbalanced; that is, they will present a less extreme perspective toward alternative or conventional medicine.
- H5: Articles that were edited by more authors will be less unbalanced.
- H6: Articles that were mainly edited by authors whose previous perspectives were, on average, relatively incongruent with the article they worked on will be less unbalanced.
- H7: Articles that were edited by heterogeneous authors will be less unbalanced.

## Method

In this section, we describe the steps from choosing both the study material and the machine learning procedures up to the creation of the variables for the main analyses. We wanted to get a lot of data sorted into three classes representing either a neutral perspective, a conventional medicine perspective, or an alternative medicine perspective. We trained a supervised machine learner to annotate the large amount of data, for which manual categorization would have been unrealistic. To obtain the training data for machine learning, two annotators manually labeled a subset of the data. We used the data upon which both annotators agreed as our gold-standard for training and testing the machine learner. In the following sections we describe in detail these and subsequent procedures, as well as the resulting variables.

But beforehand, some ethical considerations should be noted. Our study used information written and publicly visible in the free-content Internet encyclopedia Wikipedia. Accordingly, the analyzed edits were considered as belonging to the public domain [45–48]. Wikipedia articles, edits as well as the authors’ nicknames could be openly read by everybody. Thus, the behavior did not occur in a private context [45]. In line with the requirements of the local ethics committee at our institute (Leibniz-Institut fuer Wissensmedien, Tuebingen, Germany), the dignity and the integrity of authors were not violated in any way in this study. With regard to the provided S1–S7 Files, and in order to protect the authors’ privacy and to prevent online identities’ being traced too easily, we have replaced the authors’ nicknames with file-specific and arbitrary but unique code numbers [47]. Nevertheless, for each author a dummy variable indicates whether the original name seemed to be an IP address (authors who were unregistered or not logged in) or not.

### Choosing relevant articles

We used the predefined category system of the second-largest Wikipedia version (the German-language Wikipedia) for determining articles that had the potential for controversy in the discussion of alternative versus conventional medicine. For this purpose, we selected all articles under the categories of *Alternativmedizin* (alternative medicine) and *Ernährung* (nutrition; as this topic also often deals with alternative medical approaches), along with all their sub-categories. This initial number of articles totaled 1,248. We rated them and evaluated their potential for controversy within their content on a 5-point scale, with a maximum at 5. Through this process, we identified 398 articles which contained at least some relevant controversy with a rating of 2 or more. On March 7, 2013, using the Wikipedia application programming interface (API), we extracted the history of all the 71,087 edits crawled for those 398 articles, including their particular text changes.

### The machine learning procedure

In order to obtain gold-standard data for the supervised machine learning, two independent raters manually evaluated all modifications of 5,000 randomly chosen edits. Thus, of the total data set, 5,000 edits were annotated by the raters, and 66,087 edits remained unannotated.

Each single edit in Wikipedia gets a certain revision ID and a timestamp but may contain several changes to several different parts of the article text. Every one of these changes (even within a single edit) may go in different directions regarding the controversy of conventional versus alternative medicine. Therefore, we decided to take *each changed paragraph within an edit* as a separate unit of annotation and refer to it as a *modification*. The corresponding instructions for the two raters were that they should decide for each modification whether it was in favor of the conventional medical perspective, in favor of the alternative medical perspective, or did not address this controversy at all and thus was to be rated as neutral.

However, because a majority of the modifications were just movements of text paragraphs from one location to another without any substantial change in the original content, only 4,673 edits (consisting of 28,371 modifications) were finally examined by Rater A and 4,855 edits (consisting of 29,030 modifications) by Rater B respectively. Altogether, 4,641 of the 5,000 (consisting of 28,117 modifications) edits were annotated by both raters. During the annotation process we realized that even many of the modifications that remained after removing those which consisted of simply shifting paragraphs did not contain any significant changes with regard to their content. Changes such as corrections of orthography or syntax were not interesting for us. Without these irrelevant edits, there remained 12,637 modifications rated by Rater A and 11,694 modifactions rated by Rater B. Rater A rated 77.8% of those remaining modifications as neutral, 10.1% as conventional, and 12.1% as alternative. Rater B rated 66.6% of them as neutral, 15.7% as conventional, and 17.8% as alternative. The overall Cohen’s kappa agreement of the two raters was κ = 0.56, which describes a moderate reliability (e.g., see [49], p. 165). Together with the codings from rater A and rater B, the text paragraphs which were annotated by *both* raters are given in S2 File (see S1 File for the description of the variables).

In a next step, we took data on which both raters had agreed as a *gold-labeled data set* for training and testing the machine learner. Because skewed class distribution in the training is a problem for many machine learning algorithms, we class-balanced our gold-standard set by randomly selecting 598 samples from each class (pro-conventional medicine, pro-alternative medicine, and neutral). We split this set of 1,794 modifications (598 per class) into a gold-labeled *training set* of 1,257 modifications (419 per class) and a gold-labeled *test set* of 587 modifications (179 cases per class).

We used natural language processing tools to help us identify linguistic units for feature extraction. For sentence detection and tokenization, we used the Apache OpenNLP (http://opennlp.apache.org) Sentence Detector and Tokenizer, and for part-of-speech tagging and dependency parsing the MATE Tools [50]. We then extracted and explored a range of different features, including word, stem and part-of-speech unigrams, bigrams and trigrams, but also more linguistically motivated features, such as lexical dependency pairs.

We experimented with two forms of dependency pairs, a labeled dependency triple including the dependency relation as label (form: *relation-dependentWord*.*headWord*) and a form without it (form: *dependentWord*.*headWord*). The *dependentWord* is a word in a sentence that grammatically depends on the *headWord*, and the relation is the grammatical relation between the dependent and its head (e.g., subject, object, nominal modifier). For example, in the sentence “John ate an apple”, the labeled dependencies are *SBJ-john*.*ate*, *NMOD-an*.*apple*, *OBJ-apple*.*ate*, and the special *ROOT-ate*.*root* marking the root of the dependency graph; and the unlabeled dependency pairs are: *john*.*ate*, *an*.*apple*, *apple*.*ate*, and *ate*.*root*.

To capture the difference between the *pre-edit version* A and the *post-edit version* B of the texts in a modification we considered only the features (bigram or dependency pairs) that changed during the modification. To calculate which features had changed, we used a set representation (in the mathematical sense) of the features extracted for A and B text versions. Then, features that were present in set A and not in set B were marked with a minus sign “−”and elements that were present in B but not in A were marked with a plus sign “+”. This representation was used for features that were based on a linguistic unit, that is, for word, stem, and part-of-speech ngrams and for dependency pairs. We used a binary feature representation so that the presence of a feature in a modification was marked with a 1 at the respective slot in the feature vector. Additionally, we experimented with features such as text and sentence length, differences between both text versions, and the frequency differences of some selected words and parts of speech.

It must be kept in mind that, besides the 5,000 edits scheduled to be rated by the two raters, a totality of 66,087 edits (consisted of 442,981 modifications) remained unannotated by the two raters. However, because many modifications were just relocations of text paragraphs, we cleaned out such modifications in cases (a) where the versions A and B were equal or when the modifications only consisted of irrelevant features (e.g., URLs or numbers), (b) when the size of the difference measured in Levenshtein distance was smaller than 3, or (c) when only punctuation characters (excluding quotation marks) were changed.

After the cleanup, 43,471 edits remained. They consisted of 115,425 modifications and belonged to 395 articles. These edits did *not* include the first edits in the articles, that is, the articles’ *start edits*. The first edits in a totality of 397 articles (for three articles, *only* the start edits were considered at all) were coded separately by hand and separated from all other steps. The reason for this was that the start edit of an article, as the first version of the Wikipedia article, was not comparable to all the other edits which were intended to be classified by the machine learner, at least with regard to the text length and from the perspective of the machine learner.

For machine learning, we used WEKA’s [51] implementation of Sequential Minimal Optimization (SMO) algorithm, which is a Support Vector Machine using the SMO algorithm for training. We used our training and test set to experiment with a variety of different models that represented different feature combinations. These models were first built and used for training on the gold-labeled training set, and their accuracy was then tested on the gold-labeled test set. We obtained our best results with a combination of stem unigrams, parts of speech bigrams, and a small collection of hand-crafted features including sentence length and text length differences. This model resulted in an accuracy of 66.8% on the test set. We also experimented with removing stop words but found that this reduced accuracy.

From the 115,425 modifications, the machine learner automatically classified 33,507 modifications as representing an alternative medical perspective; 23,995 modifications as representing a conventional medical perspective, and 57,923 modifications as neutral. These automatic annotations are given in S3 File (see S1 File for the description of the variables). This data file also contains the manual codings of those 198 start edits that were considered as not-neutral edits. The class distribution of the automatic coded data corresponded approximately to the class distribution of our hand-coded data (see S2 File). To verify our machine learning accuracy, a human annotator labeled 670 new randomly chosen samples of modifications. The human and the machine agreed in 61% of all cases, corresponding to a κ of 0.36 which describes a fair reliability ([49], p. 165). Together with the codings from the human rater and the machine, the text paragraphs that were annotated by both are given in S4 File (see S1 File for the description of the variables).

It should be noted at this point that even modifications that have a strong impact on the meaning of a paragraph can often be very small and quite subtle. As a consequence, they are very hard to classify. For example, the sentences “medication is effective” and “homeopathy is effective” can be changed into “medication may be effective” and “homeopathy may be effective” respectively. This same modification (“may be” instead of “is”) makes the first statement less pro-conventional medicine whereas it makes the second statement less pro-alternative medicine. So the same modification may have opposite effects with regard to the conviction that is expressed, depending on the particular combination of words in the sentences that were changed.

### Variables and units of analysis

In our further analysis, we aimed to build profiles of articles and authors representing the temporal evolution of their perspectives, for example, in order to calculate their incongruity at the time before a certain edit was made. Therefore, we combined the categorized modifications, that is, the changed and then categorized paragraphs, within each edit. In cases of edits consisting of modifications of different classes, we proceeded as follows: A combination of conventional and neutral modifications was considered to be an edit with a conventional medical perspective. Analogously, a mixture of alternative and neutral modifications was considered to be an edit with an alternative medical perspective. Finally, an edit with a combination of alternative and conventional modifications was coded with a different number expressing the average of the coefficients of the modifications, with *conventional perspective* taken as 0 and *alternative perspective* as 1. For example, an edit with two alternative and three conventional modifications would be coded as (2·1 + 3·0) ÷ (2 + 3) = 0.40. In the following text we refer to this measure that describes each edit as *edit polarity*.

Up to this point, we had 43,471 edits consisting of 115,425 automatically coded modifications from 395 articles. However, as mentioned above, for 397 articles, the start edits were coded separately and manually. These coded modifications include 140 cases representing an alternative medical perspective, 58 cases representing a conventional medical perspective, and 199 cases which were classified as neutral.

Together with the hand-coded start edits, we obtained a merged data set of 43,868 edits. Among them were 22,280 neutral edits (exclusively consisting of neutral modifications); 7,380 conventional perspective edits; 9,045 alternative perspective edits; and 5,163 mixed edits, which were on the scale somewhere between alternative and conventional. The total number of different articles to which these edits were made was 398 (394 articles with coded start edits *and* coded subsequent edits, 3 articles with coded start edits only, and 1 article with coded subsequent edits only). These edits were made by 13,508 different authors.

In further steps, we excluded edits made by bots, that is, wiki programs that make automatic article changes. As we were mainly interested in edits with some expression of the two contradictory medical perspectives, we further excluded those edits that were classified as neutral (consisting of only neutral modifications). Repeated edits in the same class that were done in a row by the same author to the same article were considered irrelevant too, because they were regarded as separately saved modifications within one single edit. After this final stage of cleaning, our *final data set* contained 389 articles (with at least one non-neutral edit) with 19,437 non-neutral edits made by 7,480 different authors (4,458 of those authors had edited anonymously).

In the time span of our study, each article, on average, contained *M* = 49.97 non-neutral edits (*SD* = 101.56), with a minimum of 1 and a maximum of 1,210 non-neutral edits. Each article was edited, in non-neutral ways, on average, from *M* = 28.07 different authors (*SD* = 44.55), with a minimum of 1 and a maximum of 384 different authors. Each author made, on average, *M* = 2.60 non-neutral edits (*SD* = 11.74), with a minimum of 1 and a maximum of 614 edits. Each author wrote, on average, in *M* = 1.46 different articles (*SD* = 2.93), with a minimum of 1 and a maximum of 102 articles.

The distribution of the automatically classified polarity in our final data set with 19,437 non-neutral edits can be characterized primarily by the high occurrence of both the polarity value of 0.00 and the polarity value of 1.00 on the conventional-alternative perspective scale. That is, most edits were either extremely pro-conventional medicine (0.00) or extremely pro-alternative medicine (1.00), with absolute values of 6,565 (33.8%) and 7,836 (40.3%) respectively. The remaining 5,036 edits (25.9%) in-between these two poles were edits with two or more modifications that were mixed in their perspectives. The mean edit polarity is near at the theoretical midpoint of the polarity scale, *M* = 0.54 (*SD* = 0.44). From these edit polarities we calculated further measures: the *article polarity*, the *article imbalance*, the *author polarity*, the *incongruity between authors and articles*, and the *heterogeneity of authors per article*.

The *article polarity* describes the perspective of an article at its current state. It is represented by the average of the polarities of all edits the article consists of (not including edits with only neutral modifications). Because edits that contain a lot of content may have a higher impact on the overall polarity of an article than edits with only a little content, we weighted the edits with the amount of change they contained (based on the corresponding non-neutral modifications). As an approximation for the amount of change, we added the sum of those linguistic tokens (e.g., words) which were present in post-edit version B but not in pre-edit version A (*added* tokens) to the sum of tokens which were present in pre-edit version A but not in post-edit version B (*deleted* tokens). We counted a certain token (e.g., a specific word) only once, that is, irrespective of how frequently or where the token appeared in both versions. In rare cases in which the total of added and deleted tokens was zero, the weights were set to 0.50 to avoid having the edit disappear completely in the averaging process. As a corresponding approximation for the start edits we counted the start edits’ unique words in the text with the help of ATLAS.ti [52].

The *article imbalance* is calculated as the absolute value of the articles’ polarity difference from the theoretical midpoint (0.50) of the scale between conventional (0.00) and alternative (1.00) medicine. Thus, an article with positioning at either 1.00 or 0.00 would have the maximum possible imbalance of 0.50.

The *author polarity* infers the perspective of an author from the previous contributions the author has made. Analogously to the article polarity, we calculated the author polarity through averaging the polarities of all edits an author had made up to a certain time point (without those edits with only neutral modifications). For this measure we did not weight the edits.

The *incongruity between an article’s and author’s perspective* was operationalized by the absolute values of the differences between the two perspectives before an edit was made.

The *heterogeneity of authors per article* was calculated by taking the root of the mean squared deviations of author values from their article-specific authors’ polarity mean within each article. Polarity values of each author per article were weighted in such a way that the totality of all values for each author had a total weight of 1 divided by the number of different authors that had contributed to this article.

## Results

In the next sections, we will provide the results of the hypothesis testing. The data files for these analyses are given in S5–S7 Files (see S1 File for the description of the variables). The main analyses were done with IBM SPSS Statistics Version 20.0 [53]. In order to be conservative rather than too liberal, we tested our one-sided hypotheses with two-sided *p*-values.

### Influence of author/article incongruity (H1)

**Fig 1:**
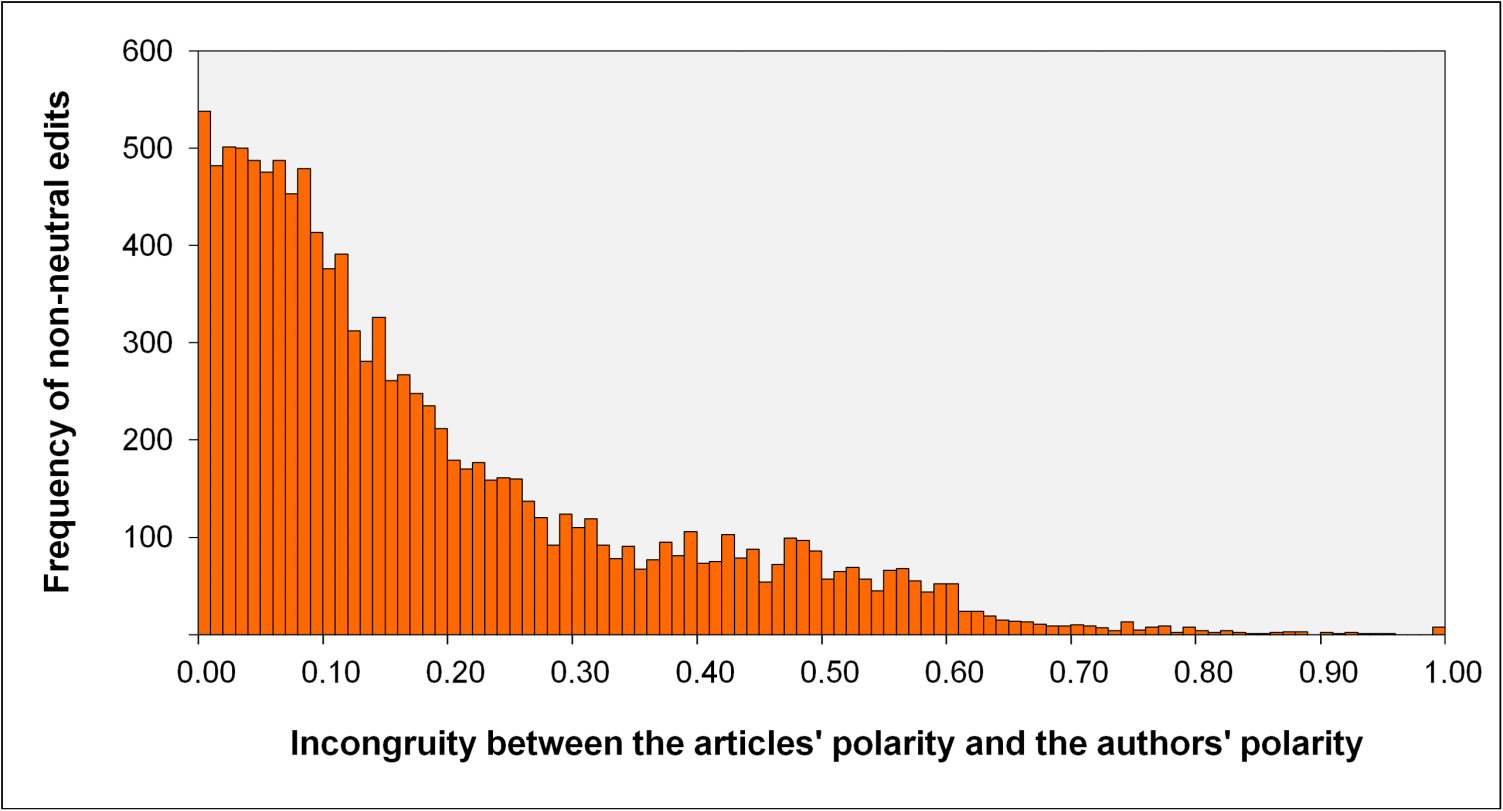
Frequencies of (non-neutral) edits as a function of the incongruity between an articles’ polarity (weighted average of all previous non-neutral edits in the article) and the polarity of the editing author (average of all non-neutral edits an author had performed before).

In H1 we expected that the incongruity between the authors’ and the articles’ current perspectives would determine the article’s development in terms of quantity of non-neutral edits, that is, there would be more non-neutral edits in articles with a medium level of incongruity. The frequency distribution of incongruity is shown in Fig 1. The figure is based on those 11,826 of the 19,437 edits for which both polarity values—those of the authors and those of the articles—were available before the corresponding edit was made. In contrast to our initial assumption in H1, Fig 1 indicates that the majority of edits showed minimal incongruity between the perspectives expressed by the authors and articles.

In order to examine if this effect was statistically significant we divided the profile positioning scale of authors and articles in five equidistant intervals. Then we performed a chi-squared test of the frequencies within the resulting cross-table. Table 1 presents the observed and the expected cell frequencies. The cells with observed cases that were higher than the expected values are marked with a plus sign. This illustrates that the main diagonal of the matrix is always overrepresented, χ^2^(16) = 436.07, *p* < .001. That is, the combination of articles and authors with similar profile positioning occurred significantly more frequently with respect to the expected baseline values. It seems that authors predominantly chose to contribute to articles with a similar positioning on the polarity scale and thus with a low incongruity to their own perspective.

**Table 1:** Observed frequencies and expected frequencies (in brackets) for the combination of articles’ polarity scores (rows) and authors’ polarity scores (columns).

	Author				
Article	0.00–0.20	0.20–0.40	0.40–0.60	0.60–0.80	0.80–1.00
0.00–0.20	42 (+)	38 (+)	61	21	7
	[14.5]	[22.7]	[82.3]	[29.8]	[19.7]
0.20–0.40	85 (+)	158 (+)	358	83	76
	[65.4]	[101.9]	[370.3]	[133.9]	[88.5]
0.40–0.60	629	1045 (+)	3780 (+)	1242	717
	[638.1]	[994.2]	[3611.8]	[1305.7]	[863.2]
0.60–0.80	225	314	1349	618 (+)	409 (+)
	[250.9]	[390.9]	[1420.3]	[513.4]	[339.4]
0.80–1.00	37	31	214	119 (+)	168 (+)
	[49.0]	[76.3]	[277.2]	[100.2]	[66.3]

χ^2^(16) = 436.07, *p* < .001. (+) Observed values that were higher than the expected values.

In sum, these results show that there is a significant influence of the incongruity between article and author, but it was not the distribution we had expected in H1. It was not a medium level of incongruity that attracted the most edits, but a low level of incongruity.

In this context, we cannot entirely disclaim the risk that there were several unregistered authors (represented as IP addresses) hiding behind the same IP. Therefore, we looked into the data for evaluating this potential danger to the data quality (see S5 File). We found that 4,458 of all 7,480 authors in S5 File could be classified as unregistered (or not logged in) users (59.6%). However, from these 4,458 unregistered authors (as the base) a total of 3,803 (85.3%) only made one non-neutral edit, 444 (10.0%) made two edits, 122 (2.7%) made three edits, 48 (1.1%) made four edits, 22 (0.5%) made five edits, and only 19 (0.4%) made more than six edits. Thus, the potential risk of severe distortion of the results caused by different authors behind the same IP address is comparatively low. In addition, a Poisson regression (robust maximum likelihood estimator) of the number of non-neutral edits on the dummy variable for unregistered (or not logged in) versus registered and logged in authors revealed a significant effect (the logarithmized quotient of the two group means), *b* = 1.30, χ^2^(1) = 316.81, *p* < .001, whereby registered authors made more non-neutral edits (*M* = 4.59, *SD* = 18.25) than unregistered or not logged in authors (*M* = 1.25, *SD* = 0.91).

### Influence of article and author on the edit bias (H2 and H3)

In H2 we assumed that the current perspective of an author would predict the perspective expressed in future edits by this author, whereby both should point in the same direction. Analogously, in H3 we expected that the current perspective of an article would predict the perspective expressed in future edits in this article.

For testing both of these hypotheses, we employed a regression model predicting the edit polarity by the articles’ polarities (derived from the previous non-neutral edits) and by the authors’ polarities (derived from the previous edits of the corresponding authors). Only those 11,826 of the 19,437 edits were considered for which both the polarity values of the authors and the polarity values of the articles were available before a certain edit was made. Besides the main effects of articles’ and authors’ polarity we also considered the potential interaction effect between the two predictors by incorporating the corresponding interaction term in the set of predictors. Before running the analysis, we centered the polarity variables at the theoretical midpoint (0.50) of the polarity scale (e.g., see [54–55]). Table 2 shows the parameter estimates of the regression analysis.

**Table 2:** Regression of the edits’ polarity on authors’ and articles’ polarity.

Variables	Estimate	*SE*	*t*-value	*p*-value	Sign.
Intercept	0.50	0.00	118.22	< .001	***
Articles’ polarity	0.13	0.03	4.48	< .001	***
Authors’ polarity	0.09	0.02	5.03	< .001	***
Interaction	-0.33	0.11	-3.06	.002	**

** *p* < .01, two-tailed.

*** *p* < .001, two-tailed.

Even though the overall *F*-value indicates statistical significance, *F*(3, 11822) = 17.12, *p* < .001, the explained variance was rather low with an estimated value for the adjusted *R*-squared smaller than .01. Thus, at this point, the high amount of statistical power has to be taken into account. Nevertheless, and in accordance with H2 and H3, there is a positive effect of both the authors’ previous polarity and the articles’ previous polarity on the polarity of the subsequent edits. However, by interpreting these main effects, the significant interaction coefficient has to be considered.

**Fig 2:**
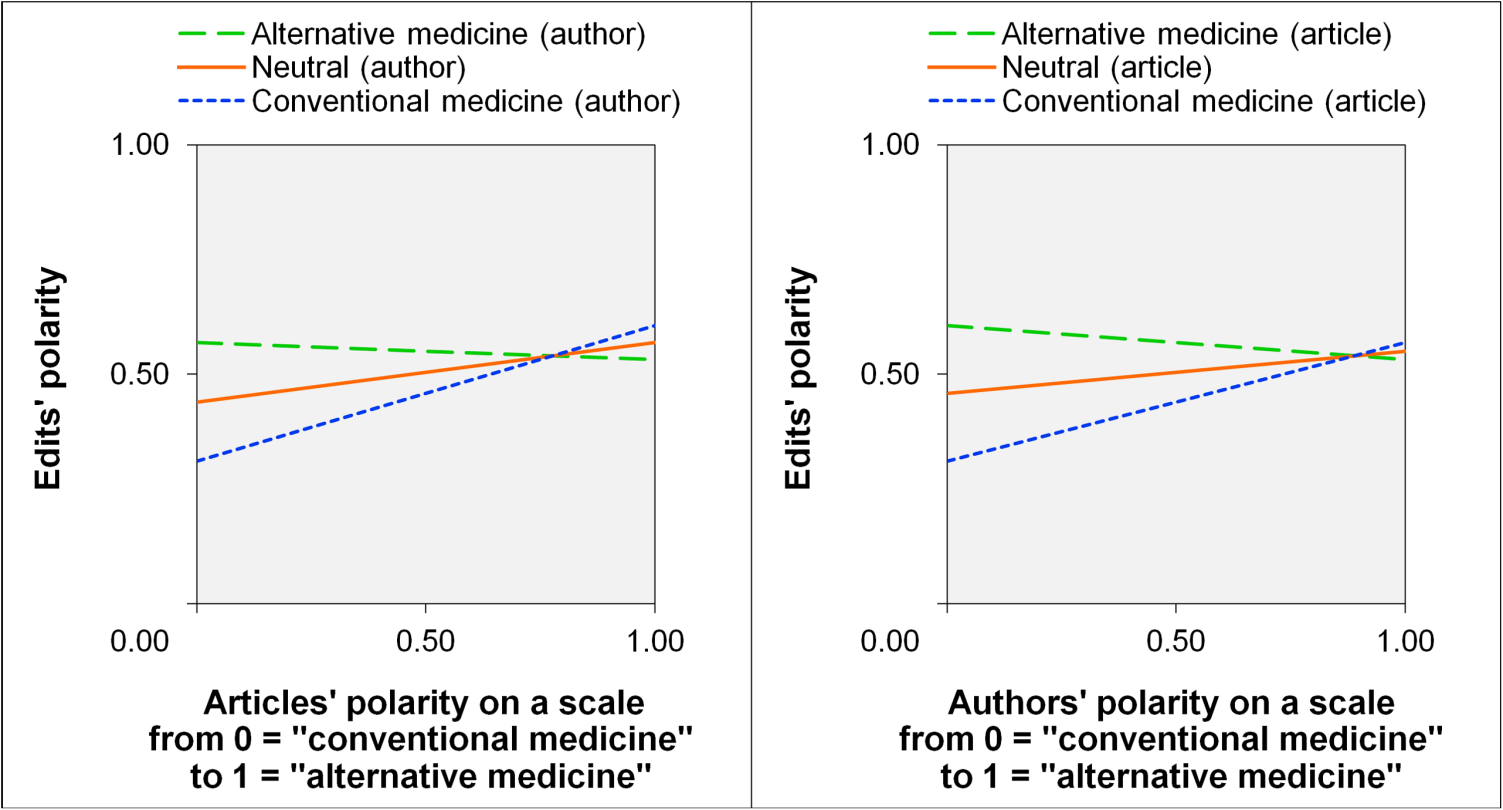
Edits’ polarity as a function of the editing author’s polarity and the article’s polarity.

Fig 2 shows (a) three conditional regression lines depicting the relationship between edit polarity and article polarity for authors with polarity values of 0.00, 0.50, and 1.00 respectively (left-hand side of Fig 2) and (b) three conditional regression lines depicting the relationship between edit polarity and author polarity for articles with polarity values of 0.00, 0.50, and 1.00 respectively (right-hand side of Fig 2). As Fig 2 indicates, the influence of the authors’ previous perspective on subsequent edits is highest for articles with a more conventional medicine perspective. That is, the difference in edit polarity between authors from both poles is highest for articles with a conventional perspective. It seems that primarily authors with a conventional perspective adapted the polarity of their edits to the article’s polarity, whereas authors with an alternative perspective did not differentiate between pro-conventional medicine and pro-alternative medicine articles. Thus, H2 (authors’ influence) holds, at least, for articles with a somewhat conventional perspective and H3 (articles’ influence) holds, at least, for authors with a more conventional perspective.

### A broad perspective on articles’ imbalance: Number of edits (H4)

**Fig 3:**
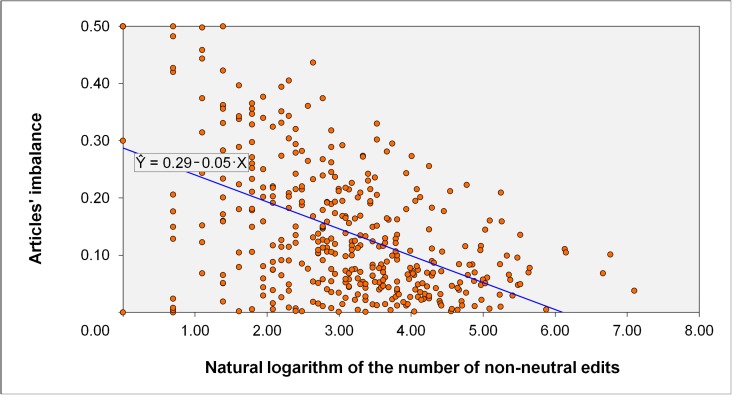
Imbalance of articles with regard to the logarithmized number of non-neutral edits an article has.

H4 assumed that articles with more non-neutral edits would be less unbalanced, that is, they would present a less extreme perspective toward alternative or conventional medicine. Fig 3 shows the relationship between the natural logarithm of the number of edits an article had already attracted and the imbalance of this article. Logarithmized values for the edit counts were used, due to the strong positive skewness of the raw data (skewness = 6.83, *SE* = 0.12) compared to the skewness of the logarithmized values (skewness = 0.03, *SE* = 0.12). With a raw mean of *M* = 49.97 (*SD* = 101.56; afterwards: *M* = 3.07, *SD* = 1.28), this transformation procedure not only seemed more appropriate than the square root transformation (resulting skewness = 2.58, *SE* = 0.12), but it also seemed justifiable against the alternative option of running a Poisson regression with the number of edits, albeit not in a causal sense, as a dependent variable (e.g., see [56]).

Remember that the article imbalance is estimated as the absolute value of the articles’ polarity difference from the theoretical midpoint (0.50) on the scale between conventional (0.00) and alternative (1.00) medicine. For the entire set of 389 articles, the correlation between the logarithm of the number of edits and the imbalance of an article is *r*(387) = -.51 and statistically highly significant, *p* < .001. The same is true if the also positively skewed variable imbalance (skewness = 1.12, *SE* = 0.12) was either log-transformed (skewness = -1.28, *SE* = 0.12), *r*(387) = -.29, *p* < .001, or square-root-transformed (skewness = 0.23, *SE* = 0.12), *r*(387) = -.44, *p* < .001. Thus, and in accordance with H4, articles with more non-neutral edits were themselves more neutral, meaning that they presented a less extreme position with regard to alternative or conventional medicine.

**Fig 4:**
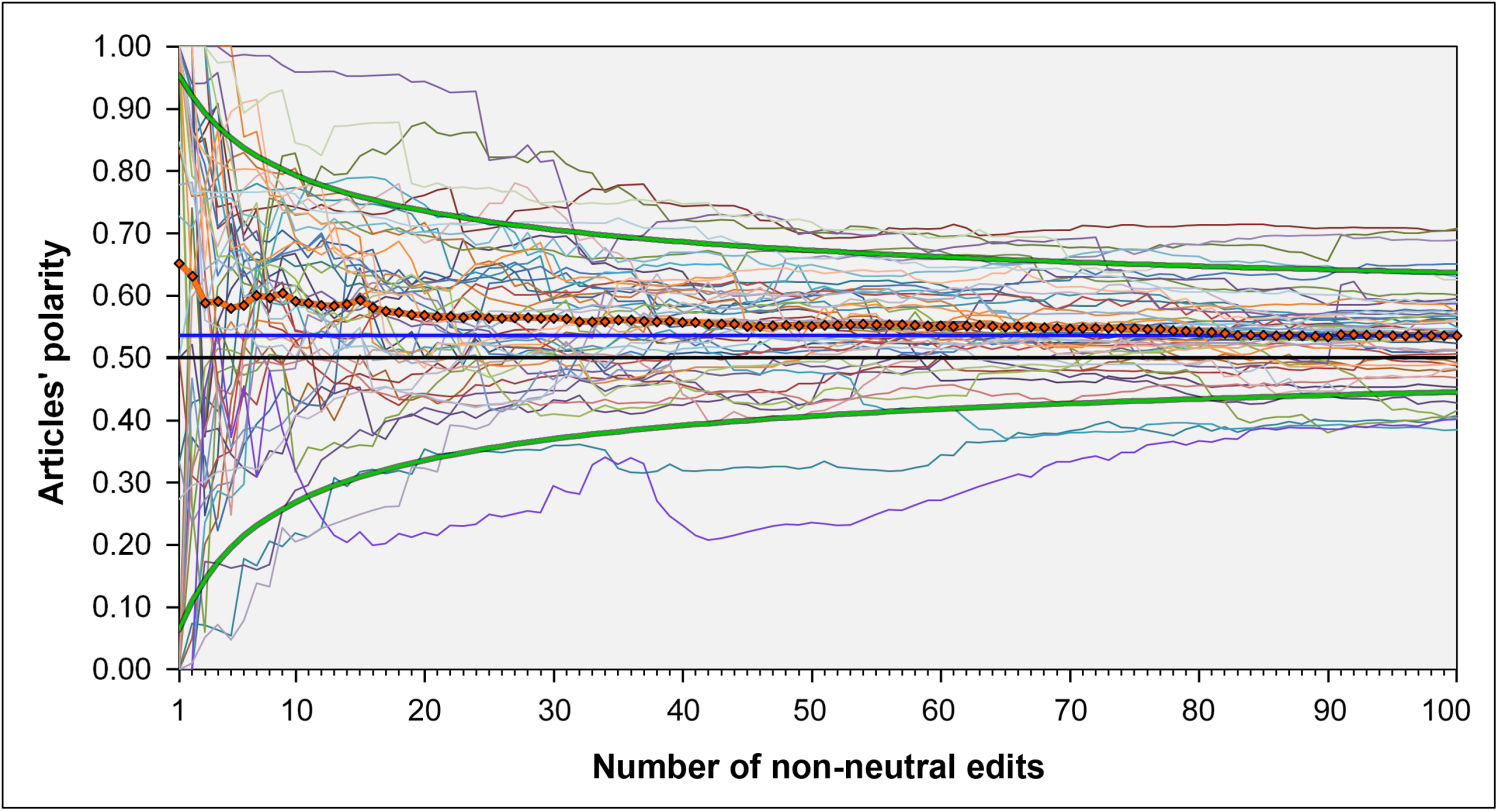
Polarity development of those *n* = 47 articles that contained at least 100 non-neutral edits. Each curve represents the polarity development of a particular article. For the 100th edit, the averaged polarity of those articles is *M* = 0.54 (*SD* = 0.08). The two bold green lines represent a 95% binomial proportion confidence interval, calculated by the method of Wilson [57] without continuity correction, with a 54 percent probability of success (e.g., observing head in a random experiment with a slightly biased coin). The bold line in black represents the theoretical midpoint (0.50) of the polarity scale. The other two bold lines represent the value 0.54 (blue) and the cross-sectional mean values (orange).

Fig 4 shows the development of the article polarity for those *n* = 47 articles that contain at least 100 non-neutral edits. This illustration makes clear that the polarity values of each article scatter less and less around the polarity value of 0.50, as an indicator for balance, as the number of edits grows. It must be mentioned, however, that also for the 100th edit, the mean value of these articles points slightly in the alternative medicine direction, *M* = 0.54 (*SD* = 0.08).

### A more finely-grained perspective on articles’ imbalance: Number of authors, heterogeneity of authors, and the incongruity between authors and articles (H5, H6, H7)

H5–H7 assumed that articles would be less unbalanced if they were edited by many authors (H5), by authors whose perspectives were relatively incongruent with the articles (H6), and by heterogeneous authors (H7). However, because many of the variables were confounded by the number of edits an article featured, we decided to take only those articles into consideration that had at least 50 non-neutral edits and to use these first 50 edits as a basis for the next steps of analysis.

Overall, there were *n* = 98 articles with at least 50 non-neutral edits. The *polarity* of those articles was calculated by averaging the polarity of these 50 edits, whereby each edit was weighted, as described above, according to the amount of change it contained. Again, the article *imbalance* was calculated by the absolute values of the differences between those polarity values and the theoretical midpoint (0.50) of the polarity scale. The *incongruity* values for each article were obtained by averaging, edit by edit, the absolute difference between an author’s polarity value (from the last point in time before the corresponding non-neutral edit was made) and the article’s polarity (from the last point in time before the corresponding non-neutral edit was made), whereby only those edits could be considered for which both an author polarity value and an article polarity value were available. That means, for example, that edits made by authors who had never made non-neutral edits before could not be considered. The same is true for the first edit of an article, because there was no article value before this specific point in time. Moreover, incongruity values of each author per article were weighted in such a way that the totality of all values for each author had a total weight of 1 divided by the number of authors relevant for the particular article. This applied, for example, if there were hypothetically only two authors A and B for an article and author A had made one edit and author B had made two edits, then the weight for the contribution of author A would be .50 and the weights for the two contributions of author B would be .25 and .25. The *heterogeneity of authors per article* was estimated by calculating the root of the mean squared deviations of author values from their article-specific mean within each article. By this method, single contributions of each author per article were weighted in the calculations in the same way as described above. And, also as before, only author polarity values could be considered that were existent before the edit was made. That means that points in time where edits were made by authors who had never made non-neutral edits before could not be considered.

In order to test H5–H7 with the 98 articles that had at least 50 non-neutral edits, we first ran a regression analysis of the article imbalance on the number of authors (H5), on the incongruity between authors and articles (H6), and on the authors’ heterogeneity (H7). However, main effects of those predictors can only be taken seriously if there were no interactions with the direction of the article polarity. This means, for example, if a predictor had a negative effect on the article imbalance regardless of the article’s polarity direction (e.g., extremely conventional or extremely alternative) then there should have been no interaction between the predictor and the direction of the polarity value behind the imbalance score of an article. Therefore, we also included a dummy variable in the regression analysis, which indicated for each article whether the article polarity differed from 0.50 in the conventional direction (value of one) or in the alternative direction (value of zero). Additionally, we included three interaction terms to represent and test the two-way interactions between the polarity direction on the one hand and the other three predictors on the other. The three continuous predictors were mean-centered before the interaction product terms were calculated (see [54–55]). With the applied parametrization, the main effects of the continuous predictors represented the conditional main effects for articles with alternative polarity directions. The interaction coefficients represented potential differences between those conditional effects and the corresponding conditional effects for articles with conventional polarity directions.

It should be noted for the dependent variable *imbalance* that modified Shapiro-Wilk-Tests [58–59] showed significant deviations from the normal distribution (*p* < .001; skewness = 1.04, *SE* = 0.24), which is not the case for the three main regressor variables (*p*s > .20). In this regard, a square root transformation of the imbalance variable (resulting skewness = 0.25, *SE* = 0.24); Shapiro-Wilk *p* = .046) could be considered to be more appropriate than a log-transformation (resulting skewness = -0.84, *SE* = 0.24; Shapiro-Wilk *p* < .001). The following analyses were therefore performed with the untransformed as well as with the square-root-transformed imbalance variable.

In a first step, we tested whether the addition of the three interaction terms significantly improved the model. This was the case for the untransformed imbalance variable with a significant improvement of the *R*^2^ value from .14 to .22, *F*(3, 90) = 2.86, *p* = .042. For the square-root-transformed imbalance, the improvement of the *R*^2^ value from .14 to .21 did not reach significance, *F*(3, 90) = 2.67, *p* = .052 (for the controversial term “marginal significance”, see, for example, [60]). Table 3 presents the results of the regression model with interaction coefficients. For the sake of completeness, in Table 3 we also included those estimates that resulted when the coding scheme for the direction variable was reversed. Table 4 presents the corresponding results for the square-root-transformed imbalance variable.

**Table 3:** Regression of articles’ imbalance on relevant predictors and their interactions with the dummy-coded direction of article polarity (estimates in parentheses result if the dummy variable for direction gets a value of zero, instead of one, for articles with pro-conventional perspectives).

Regression parameter	Estimate	*SE*	*t*-value	*p*-value	Sign.
Intercept	0.09	0.01	11.98	< .001	***
	(0.06)	(0.01)	(4.87)	(< .001)	(***)
Direction of polarity (IV0)	-0.03	0.02	-2.10	.038	*
	(0.03)	(0.02)	(2.10)	(.038)	(*)
Number of authors (IV1)	0.00	0.00	0.61	.541	ns
	(-0.00)	(0.00)	(-2.14)	(.035)	(*)
Incongruity (IV2)	0.80	0.22	3.64	< .001	***
	(0.06)	(0.33)	(0.19)	(.850)	(ns)
Authors’ heterogeneity (IV3)	-0.53	0.25	-2.11	.037	*
	(0.16)	(0.28)	(0.57)	(.573)	(ns)
Interaction: IV0 × IV1	-0.01	0.00	-2.20	.030	*
	(0.01)	(0.00)	(2.20)	(.030)	(*)
Interaction: IV0 × IV2	-0.73	0.40	-1.83	.070	ns
	(0.73)	(0.40)	(1.83)	(.070)	(ns)
Interaction: IV0 × IV3	0.69	0.37	1.84	.069	ns
	(-0.69)	(0.37)	(-1.84)	(.069)	(ns)

If the dummy variable for direction gets a value of zero (instead of one) for pro-alternative medicine articles, the coefficients of the three continuous predictors represent the conditional effects of those variables within the pro-alternative medicine articles. If the dummy variable gets a value of zero for pro-conventional medicine articles, the coefficients of the other predictors represent the conditional effects of those variables within the pro-conventional medicine articles. Values of the interaction coefficients represent the difference between the two conditional effects of the corresponding continuous predictor. ns = not significant.

* p < .05, two-tailed.

*** *p* < .001, two-tailed.

**Table 4:** Regression of articles’ imbalance (square-root-transformed) on relevant predictors and their interactions with the dummy-coded direction of article polarity (estimates in parentheses result if the dummy variable gets a value of zero for conventional perspectives).

Regression parameter	Estimate	*SE*	*t*-value	*p*-value	Sign.
Intercept	0.28	0.01	21.19	< .001	***
	(0.23)	(0.02)	(10.39)	(< .001)	(***)
Direction of polarity (IV0)	-0.06	0.03	-2.21	.029	*
	(0.06)	(0.03)	(2.21)	(.029)	(*)
Number of authors (IV1)	0.00	0.00	0.39	.697	ns
	(-0.01)	(0.00)	(-2.20)	(.030)	(*)
Incongruity (IV2)	1.29	0.37	3.47	< .001	***
	(0.26)	(0.57)	(0.45)	(.652)	(ns)
Authors’ heterogeneity (IV3)	-0.92	0.43	-2.14	.035	*
	(0.25)	(0.47)	(0.53)	(.598)	(ns)
Interaction: IV0 × IV1	-0.01	0.00	-2.16	.033	*
	(0.01)	(0.00)	(2.16)	(.033)	(*)
Interaction: IV0 × IV2	-1.03	0.68	-1.52	.132	ns
	(1.03)	(0.68)	(1.52)	(.132)	(ns)
Interaction: IV0 × IV3	1.16	0.64	1.83	.070	ns
	(-1.16)	(0.64)	(-1.83)	(.070)	(ns)

If the dummy variable for direction gets a value of zero (instead of one) for pro-alternative medicine articles, the coefficients of the three continuous predictors represent the conditional effects of those variables within the pro-alternative medicine articles. If the dummy variable gets a value of zero for pro-conventional medicine articles, the coefficients of the other predictors represent the conditional effects of those variables within the pro-conventional medicine articles. Values of the interaction terms represent the difference between the two conditional effects of the corresponding continuous predictor. ns = not significant.

* p < .05, two-tailed.

*** *p* < .001, two-tailed.

With the initially applied dummy coding of the direction variable (value of zero for alternative medicine), it seems, at least if all other predictors were fixed on their mean values, that the articles’ imbalance was higher for articles with a more alternative perspective. This is reflected in both Table 3 (untransformed imbalance) and Table 4 (square-root-transformed imbalance).

Moreover, the conditional effects for articles with a more alternative perspective indicated that there was no influence of the number of authors, a positive effect of incongruity, and a negative effect of the authors’ heterogeneity. That is, a pro-alternative medicine article was more extreme if the incongruity between authors and an article was high and if the heterogeneity between these authors was low (see Tables 3 and 4). However, for articles with a rather conventional perspective (values in parentheses in Tables 3 and 4), there was a negative effect of the number of authors, a non-significant effect of incongruity, and a non-significant effect of heterogeneity.

Thus, H5 that postulated a negative influence of the number of authors on the article imbalance was only supported for articles with a conventional perspective. H6 that postulated a *negative* influence of incongruity on the article imbalance had to be rejected for both kinds of articles. H7 that assumed a negative influence of authors’ heterogeneity on the article imbalance was only supported for articles with an alternative perspective. Together with the fact of a significant positive correlation between articles’ polarity and articles’ imbalance, *r*(96) = .46, *p* < .001, these results could be taken as an argument against the comparability between the two poles of the polarity scale. Moreover, these results could primarily be seen as a challenge to the bi-polarity concept of the polarity scale.

However, although the regression coefficients seem to be different for both kinds of articles, there was a significant interaction with the direction of the article polarity (*p* < .05) only for the number of authors, and therefore only for one of the three continuous predictors. Nevertheless and in an exploratory sense, it seems to be wise not to ignore those interaction coefficients completely. At least because the null hypothesis “there is no statistical interaction” is the preferred hypothesis here and as such, an (a priori) significance level of .10 or .20 is not unusual (e.g., [61]) but cannot be applied here as an a priori significance level (e.g., see [62]).

Another complexity appears if the conditional coefficients from the multiple regression analyses were compared to coefficients which resulted from analyses where only one of the three continuous predictors was included together with the articles’ direction and the corresponding interaction term. The resulting coefficients are shown in S1 and S2 Tables. Comparisons of S1 and S2 Tables with the results from Tables 3 and 4 show that, for pro-alternative medicine articles, the coefficient for heterogeneity was negative when considering the other two continuous predictors, and it was positive (but not significant) when the other two predictors were not included in the analyses. At this point we should take a look at the zero-order correlation coefficients shown in the S3 Table.

It is obvious in the S3 Table, especially with regard to pro-alternative medicine articles, that the two predictors *incongruity* and *heterogeneity* have a strong linear relationship. This means, the greater the average incongruity between authors and an article the greater the variation among the authors of that article. Furthermore, because incongruity has a positive effect on imbalance, it seems, when heterogeneity is considered separately, that heterogeneity also has a positive effect on imbalance. However, when incongruity additionally is taken into account, a negative partial effect of heterogeneity on imbalance appears. Thus, at least with regard to pro-alternative medicine articles, heterogeneity has a negative effect on imbalance when incongruity is held constant, that is, for a fixed incongruity value. Finally, it should be noted that step-down testing procedures starting from quasi-saturated regression models (contain all possible interaction terms; see [54]) reveal that neither the omission of the four-way interaction term, nor the omission of all four three-way interaction terms, nor the omission of all six two-way interactions result in significant decreases in *R*^2^ values (see S4 and S5 Tables). Accordingly, S4 and S5 Tables provide no indications of statistical interactions between incongruity and heterogeneity.

## Discussion and conclusions

In this study we investigated factors assumed to influence the development of Wikipedia articles around the controversial topic of alternative medicine. The result pattern was mixed in terms of support of our hypotheses. First of all, and against our expectations in H1, it was not a medium level of incongruity that attracted authors to edit an article. Rather, most non-neutral edits were made if the incongruity between an author’s perspective and an article’s perspective was low. With regard to H2, the author’s preliminary perspective played the expected role of predicting resulting edits only insofar as the author’s perspective resulted in edit-related differences when the article’s perspective was pro-conventional medicine. Similarly, the article’s previous perspective played the role expected in H3 only insofar as this perspective resulted in edit-related differences when the author’s perspective was pro-conventional medicine. Thus, there is support for hypotheses H2 and H3, but it is limited to certain circumstances. By investigating the articles’ imbalance, that is, how extreme they were on the polarity scale, we found that a higher number of non-neutral edits was accompanied by a lower level of imbalance. This supported H4 that had postulated a negative relationship between the number of non-neutral edits and an article’s imbalance.

Findings on the influence of the number of authors (H5), of the author-article incongruity (H6), and of the authors’ heterogeneity (H7) were again mixed. Regression analyses revealed the expected negative influence on articles’ imbalance only for the number of authors (H5) and for heterogeneity of authors (H7). However, the finding for number of authors was only valid for pro-conventional medicine articles and the finding for heterogeneity of authors was only valid for pro-alternative medicine articles. Finally, and against H6 that had postulated a negative influence of incongruity on imbalance, we found a significant positive effect of incongruity within the pro-alternative medicine articles.

Nevertheless, a significant interaction with the direction of the article polarity was found only for one of the three continuous predictors. This could be a hint that the statistical power was too small to find consistent result patterns (e.g., [54–55]). Furthermore, a prerequisite for detecting the conditional negative effect of heterogeneity on imbalance was to use incongruity as an additional predictor. Without taking incongruity into account, the effect of heterogeneity seems to be positive. Aside from empirical random fluctuations, such a phenomenon could be associated with the Simpson's paradox for continuous variables [55, 63–66]). The strong positive relationship between incongruity and heterogeneity, that is, their multicollinearity, also challenges the discriminant validity of both constructs and should be circumvented in future studies.

With regard to the authors, our findings point to the importance of the authors’ previous perspectives for both the initial selection of articles, for editing them in a non-neutral direction, and for the direction as well as the polarity of the resulting edit. Whereas experimental lab studies [42–43] have shown that a medium level of incongruity between the author and an artifact was most productive for knowledge construction processes, the study presented here shows that authors in the real Wikipedia environment chose primarily articles with only a low level of incongruity for their edits. This indicates the existence of a selection bias (e.g., [22, 67–68]).

It is clear that the initial selection bias could impede efficient knowledge construction and learning processes and it could prevent reaching the full potential of knowledge construction environments such as Wikipedia. From this perspective, it seems unlikely that authors with a certain a-priori polarity will come in contact with views that are different from their own views (e.g., see [69]). However, our data do not allow us to infer whether authors only consider articles with low incongruity. A more optimistic view could be that individuals receive information from a broad spectrum of articles with different polarities but assume the author role only for those articles for which they see themselves as experts. This, however, does not rule out the possibility that a “spiral of silence” [70] could take over control whereby the diversity of perspectives in the artifact would disappear, or not even arise (e.g., [71]).

Authors did not only choose articles for which the incongruity was low, the polarity of the subsequent non-neutral edit was also influenced by the author’s preexisting polarity. However, the latter finding seems to be reserved to and was mostly obvious for articles with a more pro-conventional medicine perspective. Potentially, such pro-conventional medicine articles, even though they do belong to the corpus of articles about alternative medicine, dedicate more space to the authors to edit them in accordance with their own perspective. On the other hand, a post-hoc explanation for the unexpected positive effect of incongruity on imbalance within the pro-alternative medicine articles could be that especially the highly unbalanced pro-alternative medicine articles attract the attention not only of very highly pro-alternative-minded authors, but also of a few opposite-minded (pro-conventional) authors. Here it should also be noted that higher imbalance in an article was mostly accompanied by a rather pro-alternative medicine perspective. This raises questions about the comparability of the two poles of the polarity scale.

Another important result was that the more an article is edited the more it will become balanced. Additionally, each edit in Wikipedia must conform with the Wikipedia NPOV policy, and with the strict and fast application of its rules Wikipedia achieves impressive outcomes with regard to knowledge construction processes (e.g., see [4]). A growing number of non-neutral edits as well as the strict application of the norms and rules could be seen as important correcting factors that preclude a spiral of imbalance in a certain direction. At least two other correcting factors are the number of authors and the heterogeneity of authors: if an artifact is edited by many authors or by authors with different perspectives, the probability is increased that an article will remain in a reasonably balanced state or that it will reach a balanced state.

What could be the practical implications of such results patterns? In the long run, articles about very controversial topics could be permanently monitored by very sophisticated on-line classifiers. After the automatic detection of controversies (e.g., [9–15]), and in addition to the automatic or manual assignment of dispute tags, a traffic light rating system could signal to administrators, authors, and users an article’s imbalance and perhaps even the direction of the bias. In the short run, an important practical implication could be deduced from the selection bias which appeared. Potential authors could be informed about this issue as well as about related psychological phenomena to make them aware of and sensitive to such biases. Furthermore, potential authors could be encouraged to edit moderately incongruent articles (e.g., for digital recommender systems, see [68]). Authors could then benefit from moderate incongruity between their own attitude and the tendencies of the artifact. Positive consequences of overcoming the selection bias could be that (a) more authors and (b) more different authors would edit an article to the effect that (c) more edits would be generated per article. These factors (number of non-neutral edits, number of authors, and heterogeneity of authors) seem to be potential candidates for playing important roles in reducing an article’s imbalance.

At this point it should be noted that all the results discussed so far are based on the automatic classification of edits by the machine learning approach. In this regard it is important to remember that the agreement between the two human raters was moderate and that the agreement between a human rater and the machine was fair for a random sample of edits. Thus, all result patterns should be regarded as preliminary, their limited causal interpretability (e.g., with regard to confounding variables) should be taken into account, and successful replications in future studies are clearly required (see [72]).

In sum, our study gives new insights that should be replicated, broadened, and deepened in future studies. Methodologically, our study has the advantage that our data stem from an actually existing online environment. So the ecological validity of our findings is high for the topic under investigation. The result pattern depicts the knowledge construction processes in the real world. However, at the beginning of each editing activity the decision to edit or not has to be made and both a topic and an article have to be selected. Thus, our findings are only valid for those authors who decide to actually edit an article within the spectrum of alternative medicine. Further research should be conducted to investigate the replicability and the generalizability of our findings to other controversial issues, where it should be examined whether polarity should be conceptualized as a bi-polar or rather as a uni-polar construct. Such research should be flanked by lab studies in which, under controlled conditions, specific parameters of potential predictors can be varied and induced experimentally.

## Supporting information

S1 Table Regression coefficients for separate regressions of articles’ imbalance (untransformed) on the following regressors: the direction of the articles’ perspective, one of the other three predictors, and the corresponding two-way interaction term. (PDF)

S2 Table Regression coefficients for separate regressions of articles’ imbalance (square-root-transformed) on the following regressors: the direction of the articles’ perspective, one of the other three predictors, and the corresponding interaction term. (PDF)

S3 Table Zero-order correlations among the variables imbalance (untransformed and square-root-transformed), number of authors, incongruity, and heterogeneity. (PDF)

S4 Table Regression of articles’ imbalance on the relevant predictors and on the two-way, three-way, and four-way interaction terms. (PDF)

S5 Table Regression of articles’ imbalance (square-root-transformed) on the relevant predictors and on the two-way, three-way, and four-way interaction terms. (PDF)

S1 File Information about the variables in S2–S7 Files. (DAT)

S2 File Modifications which were annotated by both human raters. (CSV)

S3 File Automatic annotations by the machine learner. (DAT)

S4 File Comparison between a human rater and the machine. (CSV)

S5 File Data for the main analyses: Edit level (H1–H3). (DAT)

S6 File Data for the main analyses: Article level A (H4). (DAT)

S7 File Data for the main analyses: Article level B (H5–H7). (DAT)
