# Supplementary material for: A productive clash of perspectives? The interplay between articles’ and authors’ perspectives and their impact on Wikipedia edits in a controversial domain
Source: PLoS One. 2017 Jun 2;12(6):e0178985. doi: 10.1371/journal.pone.0178985 (PMC5456356; doi:10.1371/journal.pone.0178985)
Supplement: S1 Table — (PDF) [file pone.0178985.s001.pdf]

S1 Table. Regression coefficients for separate regressions of articles' imbalance (untransformed) on the following regressors: the direction of the articles' perspective, one of the other three predictors, and the corresponding two-way interaction term.

Regression parameter	Estimate	*SE*	*t*-value	*p*-value	Sign.
Direction of polarity (IV0)<br>together with IV1	-0.03<br>(0.03)	0.02<br>(0.02)	-2.12<br>(2.12)	.037<br>(.037)	*<br>(*)
Direction of polarity (IV0)<br>together with IV2	-0.02<br>(0.02)	0.01<br>(0.01)	-1.58<br>(1.58)	.118<br>(.118)	ns<br>(ns)
Direction of polarity (IV0)<br>together with IV3	-0.03<br>(0.03)	0.02<br>(0.02)	-1.63<br>(1.63)	.105<br>(.105)	ns<br>(ns)
Number of authors (IV1)	0.00<br>(-0.00)	0.00<br>(0.00)	1.22<br>(-2.20)	.227<br>(.031)	ns<br>(*)
Incongruity (IV2)	0.47<br>(0.31)	0.15<br>(0.31)	3.15<br>(1.00)	.002<br>(.318)	**<br>(ns)
Authors' heterogeneity (IV3)	0.16<br>(0.20)	0.18<br>(0.28)	0.86<br>(0.69)	.391<br>(.489)	ns<br>(ns)
Interaction: IV0 × IV1	-0.01<br>(0.01)	0.00<br>(0.00)	-2.51<br>(2.51)	.014<br>(.014)	*<br>(*)
Interaction: IV0 × IV2	-0.16<br>(0.16)	0.35<br>(0.35)	-0.46<br>(0.46)	.648<br>(.648)	ns<br>(ns)
Interaction: IV0 × IV3	0.04<br>(-0.04)	0.34<br>(0.34)	0.11<br>(-0.11)	.911<br>(.911)	ns<br>(ns)

Note. Estimates without parentheses result if the dummy variable for articles' perspectives gets a value of zero for articles with an alternative perspective and a value of one for articles with a conventional perspective. Estimates in parentheses result if the dummy variable gets a value of zero for articles with a conventional perspective and a value of one for articles with an alternative perspective.

* $p < .05$, two-tailed. ** $p < .01$, two-tailed. ns = not significant.
