# Supplementary material for: A productive clash of perspectives? The interplay between articles’ and authors’ perspectives and their impact on Wikipedia edits in a controversial domain
Source: PLoS One. 2017 Jun 2;12(6):e0178985. doi: 10.1371/journal.pone.0178985 (PMC5456356; doi:10.1371/journal.pone.0178985)
Supplement: S2 Table — (PDF) [file pone.0178985.s002.pdf]

S2 Table. Regression coefficients for separate regressions of articles' imbalance (square-root-transformed) on the following regressors: the direction of the articles' perspective, one of the other three predictors, and the corresponding interaction term.

**Regression parameter**	**Estimate**	***SE***	***t*-value**	***p*-value**	**Sign.**
Direction of polarity (IV0)<br>together with IV1	-0.06<br>(0.06)	0.03<br>(0.03)	-2.28<br>(2.28)	.025<br>(.025)	*<br>(*)
Direction of polarity (IV0)<br>together with IV2	-0.04<br>(0.04)	0.03<br>(0.03)	-1.66<br>(1.66)	.100<br>(.100)	ns<br>(ns)
Direction of polarity (IV0)<br>together with IV3	-0.05<br>(0.05)	0.03<br>(0.03)	-1.74<br>(1.74)	.085<br>(.085)	ns<br>(ns)
Number of authors (IV1)	0.00<br>(-0.01)	0.00<br>(0.00)	0.97<br>(-2.35)	.335<br>(.021)	ns<br>(*)
Incongruity (IV2)	0.72<br>(0.69)	0.26<br>(0.53)	2.84<br>(1.29)	.006<br>(.200)	**<br>(ns)
Authors' heterogeneity (IV3)	0.20<br>(0.36)	0.31<br>(0.48)	0.64<br>(0.75)	.522<br>(.454)	ns<br>(ns)
Interaction: IV0 × IV1	-0.01<br>(0.01)	0.00<br>(0.00)	-2.54<br>(2.54)	.013<br>(.013)	*<br>(*)
Interaction: IV0 × IV2	-0.04<br>(0.04)	0.59<br>(0.59)	-0.07<br>(0.07)	.948<br>(.948)	ns<br>(ns)
Interaction: IV0 × IV3	0.16<br>(-0.16)	0.57<br>(0.57)	0.28<br>(-0.28)	.781<br>(.781)	ns<br>(ns)

Note. Estimates without parentheses result if the dummy variable for articles' perspectives gets a value of zero for articles with an alternative perspective and a value of one for articles with a conventional perspective. Estimates in parentheses result if the dummy variable gets a value of zero for articles with a conventional perspective and a value of one for articles with an alternative perspective.

* $p < .05$, two-tailed. ** $p < .01$, two-tailed. ns = not significant.
