# Supplementary material for: A productive clash of perspectives? The interplay between articles’ and authors’ perspectives and their impact on Wikipedia edits in a controversial domain
Source: PLoS One. 2017 Jun 2;12(6):e0178985. doi: 10.1371/journal.pone.0178985 (PMC5456356; doi:10.1371/journal.pone.0178985)
Supplement: S3 Table — (PDF) [file pone.0178985.s003.pdf]

S3 Table. Zero-order correlations among the variables imbalance (untransformed and square-root-transformed), number of authors, incongruity, and heterogeneity.

	1	2	3	4	5
**1. Articles' imbalance**	—	.97***	.04	.32**	.11
	—	.97***	.14	.35**	.10
	—	.97***	-.44*	.20	.14
**2. Articles‘ imbalance (square root)**	.97***	—	.01	.31**	.10
	.97***	—	.11	.33**	.08
	.97***	—	-.45*	.24	.15
**3. Number of authors**	.04	.01	—	.08	.04
	.14	.11	—	.15	.06
	-.44*	-.45*	—	-.28	-.04
**4. Incongruity**	.32**	.31**	.08	—	.64***
	.35**	.33**	.15	—	.74***
	.20	.24	-.28	—	.33
**5. Authors' heterogeneity**	.11	.10	.04	.64***	—
	.10	.08	.06	.74***	—
	.14	.15	-.04	.33	—

Note. The first row for each pair of variables shows the unconditional coefficient ($df = 96$), the second row shows the conditional coefficient for those $n = 69$ articles with a pro-alternative medicine perspective ($df = 67$), and the third row shows the conditional coefficient for those $n = 29$ articles with a pro-conventional medicine perspective ($df = 27$).

* $p < .05$, two-tailed. ** $p < .01$, two-tailed. *** $p < .001$, two-tailed.
