# Supplementary material for: A productive clash of perspectives? The interplay between articles’ and authors’ perspectives and their impact on Wikipedia edits in a controversial domain
Source: PLoS One. 2017 Jun 2;12(6):e0178985. doi: 10.1371/journal.pone.0178985 (PMC5456356; doi:10.1371/journal.pone.0178985)
Supplement: S4 Table — (PDF) [file pone.0178985.s004.pdf]

S4 Table. Regression of articles' imbalance on the relevant predictors and on the two-way, three-way, and four-way interaction terms.

Regression parameter	Estimate	*SE*	*t*-value	*p*-value	Sign.
Intercept	0.09	0.01	9.46	< .001	***
Direction of polarity (IV0)	-0.03	0.02	-1.72	.089	ns
Number of authors (IV1)	0.00	0.00	0.74	.462	ns
Incongruity (IV2)	0.84	0.25	3.40	.001	**
Authors' heterogeneity (IV3)	-0.56	0.28	-2.04	.045	*
Interaction: IV0 × IV1	-0.01	0.00	-2.24	.028	*
Interaction: IV0 × IV2	-0.63	0.49	-1.29	.201	ns
Interaction: IV0 × IV3	0.80	0.43	1.84	.069	ns
Interaction: IV1 × IV2	-0.01	0.03	-0.54	.590	ns
Interaction: IV1 × IV3	0.02	0.04	0.54	.590	ns
Interaction: IV2 × IV3	1.31	2.96	0.44	.659	ns
Interaction: IV1 × IV2 × IV3	-0.09	0.39	-0.23	.819	ns
Interaction: IV0 × IV2 × IV3	3.27	8.61	0.38	.705	ns
Interaction: IV0 × IV1 × IV3	-0.04	0.08	-0.46	.644	ns
Interaction: IV0 × IV1 × IV2	0.04	0.07	0.66	.510	ns
Interaction: IV0 × IV1 × IV2 × IV3	1.10	1.22	0.90	.370	ns

Note. R^2 values before and after the four-way interaction term was dropped from the quasi-saturated model: .23 and .23, $F(1, 82) = 0.81$, $p = .370$. R^2 values before and after the four three-way interaction terms were dropped from the model which also contains all possible lower-order terms: .23 and .22, $F(4, 83) = 0.14$, $p = .969$. R^2 values before and after the six two-way interaction terms were dropped from the model which also contains the main effects: .22 and .14, $F(6, 87) = 1.51$, $p = .185$.

* $p < .05$, two-tailed. ** $p < .01$, two-tailed. *** $p < .001$, two-tailed. ns = not significant.
