# Supplementary material for: A productive clash of perspectives? The interplay between articles’ and authors’ perspectives and their impact on Wikipedia edits in a controversial domain
Source: PLoS One. 2017 Jun 2;12(6):e0178985. doi: 10.1371/journal.pone.0178985 (PMC5456356; doi:10.1371/journal.pone.0178985)
Supplement: S5 Table — (PDF) [file pone.0178985.s005.pdf]

S5 Table. Regression of articles' imbalance (square-root-transformed) on the relevant predictors and on the two-way, three-way, and four-way interaction terms.

**Regression parameter**	**Estimate**	***SE***	***t*-value**	***p*-value**	**Sign.**
Intercept	0.28	0.02	16.92	< .001	***
Direction of polarity (IV0)	-0.05	0.03	-1.70	.094	ns
Number of authors (IV1)	0.00	0.00	0.48	.632	ns
Incongruity (IV2)	1.39	0.42	3.30	.001	**
Authors' heterogeneity (IV3)	-0.99	0.47	-2.11	.038	*
Interaction: IV0 × IV1	-0.01	0.00	-1.91	.059	ns
Interaction: IV0 × IV2	-0.66	0.84	-0.79	.430	ns
Interaction: IV0 × IV3	1.18	0.73	1.61	.111	ns
Interaction: IV1 × IV2	-0.03	0.05	-0.66	.510	ns
Interaction: IV1 × IV3	0.03	0.07	0.43	.670	ns
Interaction: IV2 × IV3	2.86	5.02	0.57	.570	ns
Interaction: IV1 × IV2 × IV3	-0.13	0.66	-0.20	.842	ns
Interaction: IV0 × IV2 × IV3	7.85	14.62	0.54	.593	ns
Interaction: IV0 × IV1 × IV3	-0.05	0.13	-0.37	.713	ns
Interaction: IV0 × IV1 × IV2	0.13	0.11	1.15	.253	ns
Interaction: IV0 × IV1 × IV2 × IV3	1.03	2.07	0.50	.621	ns

Note. R^2 values before and after the four-way interaction term was dropped from the quasi-saturated model: .23 and .23, $F(1, 82) = 0.25$, $p = .621$. R^2 values before and after the four three-way interaction terms were dropped from the model which also contains all possible lower-order terms: .23 and .22, $F(4, 83) = 0.38$, $p = .820$. R^2 values before and after the six two-way interaction terms were dropped from the model which also contains the main effects: .22 and .14, $F(6, 87) = 1.42$, $p = .216$.

* $p < .05$, two-tailed. ** $p < .01$, two-tailed. *** $p < .001$, two-tailed. ns = not significant.
